# Splenectomy Improves Hemostatic and Liver Functions in Hepatosplenic Schistosomiasis Mansoni

**DOI:** 10.1371/journal.pone.0135370

**Published:** 2015-08-12

**Authors:** Luiz Arthur Calheiros Leite, Adenor Almeida Pimenta Filho, Rita de Cássia dos Santos Ferreira, Caíque Silveira Martins da Fonseca, Bianka Santana dos Santos, Silvia Maria Lucena Montenegro, Edmundo Pessoa de Almeida Lopes, Ana Lúcia Coutinho Domingues, James Stuart Owen, Vera Lucia de Menezes Lima

**Affiliations:** 1 Departamento de Bioquímica, Centro de Ciências Biológicas, Universidade Federal de Pernambuco, Recife, Pernambuco, Brazil; 2 Departamento de Biofísica e Radiobiologia, Universidade Federal de Pernambuco, Recife, Pernambuco, Brazil; 3 Departamento de Medicina Tropical, Centro de Ciências da Saúde, Universidade Federal de Pernambuco, Recife, Brazil; 4 Departamento de Imunologia, Centro de Pesquisa Aggeu Magalhães (CPqAM)/FIOCRUZ–Recife, Pernambuco, Brazil; 5 Departamento de Medicina Clínica, Centro de Ciências da Saúde, Hospital das Clínicas, Universidade Federal de Pernambuco, Recife, Pernambuco, Brazil; 6 Institute of Liver and Digestive Health, Division of Medicine, University College London Medical School, Royal Free Campus, London, United Kingdom; University Hospital Oldenburg, GERMANY

## Abstract

**Background:**

Schistosomiasis mansoni is a chronic liver disease, in which some patients (5–10%) progress to the most severe form, hepatosplenic schistosomiasis. This form is associated with portal hypertension and splenomegaly, and often episodes of gastrointestinal bleeding, even with liver function preserved. Splenectomy is a validated procedure to reduce portal hypertension following digestive bleeding. Here, we evaluate beneficial effects of splenectomy on blood coagulation factors and liver function tests in hepatosplenic schistosomiasis mansoni compared to non-operated patients.

**Methodology/Principal Findings:**

Forty-five patients who had undergone splenectomy surgery were assessed by laboratory analyses and ultrasound examination and compared to a non-operated group (n = 55). Blood samples were obtained for liver function tests, platelet count and prothrombin time. Coagulation factors (II, VII, VIII, IX and X), protein C and antithrombin IIa, plasminogen activator inhibitor-1 were measured by routine photometric, chromogenic or enzyme-linked immunosorbent assays, while hyperfibrinolysis was defined by plasminogen activator inhibitor-1 levels. Both groups had similar age, gender and pattern of periportal fibrosis. Splenectomized patients showed significant reductions in portal vein diameter, alkaline phosphatase and bilirubin levels compared to non-operated patients, while for coagulation factors there were significant improvement in prothrombin, partial thromboplastin times and higher levels of factor VII, VIII, IX, X, protein C and plasminogen activator inhibitor-1.

**Conclusion/Significance:**

This study shows that the decrease of flow pressure in portal circulation after splenectomy restores the capacity of hepatocyte synthesis, especially on the factor VII and protein C levels, and these findings suggest that portal hypertension in patients with hepatosplenic schistosomiasis influences liver functioning and the blood coagulation status.

## Introduction

Schistosomiasis causes one of the most prevalent liver diseases, affecting more than 200 million people in over 74 different countries and is a major public health problem in the Northeast region of Brazil [1–2]. Nearly 10% of patients infected by *Schistosoma mansoni* progress to the most severe form, hepatosplenic (HS) schistosomiasis, which is characterized by periportal fibrosis (PPF), obstruction by eggs of intrahepatic veins, presinusoidal portal hypertension, splenomegaly, hemodynamic and lipid abnormalities, frequently resulting in upper digestive bleeding [3–5].

Upon blocking the terminal branches of the portal vein, the deposition of numerous *S*. *mansoni* eggs provokes granulomatous reactions with subsequent fibrosis, intrahepatic portal vein obstruction and increased resistance of blood flow to the sinusoids [6]. Splenomegaly results from the congestion caused by egg obstruction and fibrosis and also from hyperplasia of cells of the reticuloendothelial system, induced by immunological stimulation due to antigens released by the worms and eggs [7–9]. In addition, it has been reported that splenomegaly leads to thrombocytopenia, associated with hypersplenism, in more than 60% of patients with HS schistosomiasis, especially in the advanced stages of the disease; however, relatively few patients present symptoms due to hypersplenism and need surgery [10].

The increased resistance to portal inflow and the hyperflux in the spleno-portal territory due to massive splenomegaly both trigger presinusoidal portal hypertension [11–13]. In schistosomiasis mansoni, PPF forms around the portal branches, whilst maintaining the architecture of the hepatic parenchyma and a normal synthetic capacity of the hepatocytes [6,14]. Nevertheless, PPF may induce slight increases in the liver enzymes, alkaline phosphatase (ALP) and gamma glutamyl transferase (γGT) [14]. On the other hand, we previously reported that in HS schistosomiasis the common markers of liver injury, alanine aminotransferase (ALT), aspartate aminotransferase (AST), ALP, γGT and bilirubin, are all significantly higher than in a control group of uninfected individuals [15].

Patients with advanced HS schistosomiasis often have abnormalities in hemostasis and fibrinolysis. These include prolongation of prothrombin time (PT), partial thromboplastin time (PPT), thrombin time (TT) as well as thrombocytopenia, hypofibrinogenemia and decreases of vitamin-K-dependent factors, which relate to the low degree of disseminated intravascular coagulation [14–17]. Moreover, some patients with HS schistosomiasis may present with hyperfibrinolysis and a consequent tendency for bleeding; levels of D-dimer and tissue plasminogen activator are increased, while plasminogen activator inhibitor-1 (PAI-1) is reduced [18].

Splenectomy with ligation of the left gastric vein and esophagogastric disconnection has become a good therapeutic option to reduce portal hypertension after episodes of gastrointestinal bleeding [8,13]. Nevertheless, 13 to 53% of patients with HS schistosomiasis develop portal vein thrombosis following this procedure [13,19]. To better understand this complication, the present study has compared serum levels of liver enzymes and the hemostatic profiles in patients with HS schistosomiasis, one group having undergone surgical splenectomy and the other being non-operated.

## Materials and Methods

### Ethical Statement

Each patient received an explanation of the study and signed a free and informed consent form. The study was approved by the Ethics Committee for Research on Humans at the Federal University of Pernambuco, Brazil (Number 028/11), in accordance with the Helsinki Declaration of 1975.

### Patients

One hundred patients with HS schistosomiasis, 45 that had been splenectomized and 55 non-operated, were consecutively selected during attendance at the outpatient clinic of the Gastroenterology Division, Hospital das Clínicas of the Federal University of Pernambuco, Recife, Brazil, between April 2011 and December 2012. All patients had been previously treated with praziquantel (50 mg/kg) at least 6 months before enrolment in the study.

The diagnosis of schistosomiasis was based on clinical history, earlier contact with water bodies in the endemic zone, history of positive parasitology for *S*. *mansoni*, specific treatment and ultrasound examination revealing PPF. Abdominal ultrasound was performed by a single researcher (ALCD) through the Acuson X 150 device, with a 3.5 mHz convex transducer (Siemens), for diagnosis, to classify the different patterns of PPF and to exclude other liver diseases such as steatosis and cirrhosis. The Niamey classification of PPF was used: pattern D (central or moderate fibrosis), pattern E (advanced fibrosis) and pattern F (very advanced fibrosis) [20–21].

Patients were not included in the study groups if they reported alcohol abuse (>60 g/day of ethanol for men and >40 g/day for women), pregnancy, diabetes mellitus, hepatitis B or C, fatty liver diseases, cirrhosis, collagenosis, chronic lymphoproliferative diseases, or any use of hepatotoxic, antiplatelet or anticoagulant drugs. A transfusion of blood within 90 days of data collection also constituted an exclusion factor. All patients were tested for markers of hepatitis B virus (HBsAg and anti-HBc), hepatitis C (anti-HCV) and HIV (anti-HIV).

### Collection and Processing of Samples

Venous blood samples were collected aseptically with minimal stasis using vacuum tubes (Vacutainer, Becton Dickinson, USA) into three tubes. The first, containing 0.106 M trisodium citrate (1:9 to blood), was for blood coagulation tests, the second without anticoagulant was for liver function tests, including AST, ALT, ALP, γGT, bilirubins and albumin, while the third tube with 0.562M ethylenediaminetetraacetic acid (EDTA-K3) was used for platelet quantification. The first two blood samples were centrifuged for 10 min at 2000 g and the plasma and serum divided into 0.5 mL aliquots and stored at -80°C until assayed.

### Biochemical and Coagulation Analysis

The serum concentration of each enzyme was divided by the upper normal value according to gender (for women and men, respectively, AST 31 and 35 U/L; ALT 31 and 41 U/L; γGT 38 and 55 U/L; and ALP 128 and 141 U/L) and expressed as the resulting ratio. Bilirubin (total, direct and indirect) and albumin were measured as μmol/L and g/L, respectively. All liver function tests were measured by automated spectrophotometry (6000 analyzer series Cobas, Roche, USA). HBsAg, anti-HBc, anti-HCV and anti-HIV markers were detected by Chemiluminescence Microparticle Immuno Assay (CeMIA) using the ARCHITECT i2000 automatic light detector and test reagents (Abbott, North Chicago, USA) to exclude enrolment of patients with hepatitis B or C, and immunodeficiency.

The platelet counts were measured by electrical impedance (Pentra DF 120, HORIBA ABX SAS Diagnostics, Brazil). Coagulation tests were performed by the chromogenic method using a Destiny Plus automatic analyzer (Trinity Biotech, Ireland) and included the determination of PT, PTT, TT and fibrinogen; coagulation factors (II, VII, VIII, IX, X), protein C and antithrombin IIa were also measured with the Destiny Plus automatic analyzer (Trinity Biotech, Ireland), while quantification of PAI-1, a measure of fibrinolysis, was by ELISA (Asserachrom Diagnostica, Stago, France).

### Statistical Analysis

Differences between continuous variables in splenectomized and non-operated HS schistosomiasis patients were compared by unpaired Student’s t test, while the Mann-Whitney test was used for comparisons of non-normally distributed variables. Continuous variables were expressed as mean ± standard error of the mean, or as median and range, while qualitative variables were expressed as absolute frequencies (percentage). The Pearson chi-square test was used to compare the different patterns of PPF. All statistical analyses were performed using StatView SAS Inc. (1998, NC, USA); P< 0.05 was considered statistically significant.

## Results

The two groups of patients with HS schistosomiasis did not differ in relation to age or gender, and had similar PPF patterns. However the mean portal vein diameter in the 55 non-operated patients was 30% greater (P<0.0001) than in the 45 patients who had undergone splenectomy (Table 1). The average time post-splenectomy was 11.5 ± 8.6 years, ranging from 2 to 33 years, with a median of 9 years.

**Table 1:** Demographic characteristics and ultrasound parameters of patients with hepatosplenic schistosomiasis who have undergone surgical splenectomy or are non-operated.

Characteristics	Hepatosplenic schistosomiasis patients	
	Non-operated	Splenectomized
Number of patients	55	45
Age (years)	50.20 ± 1.86	50.19 ± 1.41
Gender		
Male	50.9%	37.8%
Female	49.1%	62.3%
Diameter portal vein (cm)	1.28 ± 0.04	0.96 ± 0.26*
Fibrosis pattern		
D	30.9%	15.6%
E	54.6%	73.3%
F	14.5%	11.1%

Values are expressed as mean ± standard error and compared by unpaired Student’s t-test; D, moderate; E, advanced; F, very advanced fibrosis

*P<0.0001.

Serum levels of AST, ALT, γGT and albumin were not significantly different between the two patient groups. However, non-operated HS patients showed significantly increased levels of ALP and of total, direct and indirect bilirubin (Table 2).

**Table 2:** Liver function tests in patients with hepatosplenic schistosomiasis who have undergone surgical splenectomy or are non-operated.

Liver Parameters	Hepatosplenic schistosomiasis patients	
	Non-operated	Splenectomized
Number of patients	55	45
AST/ULN	1.58 ± 0.14	1.45 ± 0.09
ALT/ULN	1.40 ± 0.16	1.29 ± 0.12
ALP/ULN	1.10 ± 0.08	0.88 ± 0.06*
γ-GT/ULN	3.11 ± 0.37	3.35 ± 0.42
Albumin (g/L)	39.8 ± 0.79	40.4 ± 0.10
Total bilirubin (μmol/L)	21.03 ± 2.05	14.17± 0.66*
Direct bilirubin (μmol/L)	8,89 ± 1,36	5.71 ± 0.51*
Indirect bilirubin (μmol/L)	12.14 ± 1.19	8.48 ± 0.56*

Values are expressed as mean ± standard error, and compared by unpaired Student’s t-test; ULN, Upper Limit of Normal

*P<0.05.

Splenectomized HS schistosomiasis patients had twice the platelet count of the non-operated patients (P<0.0001); they also exhibited less prolonged PT and PTT than those of the non-operated HS group. In addition, the levels of coagulation factors VII, VIII, IX, X, and protein C were significantly higher in the splenectomized group than in the HS schistosomiasis patient group, whereas no differences were noted for TT and the levels of factor II, antithrombin IIa and fibrinogen (Table 3).

**Table 3:** Coagulation and fibrinolytic parameters in patients with hepatosplenic schistosomiasis who have undergone surgical splenectomy or are non-operated.

Coagulation tests	Hepatosplenic schistosomiasis patients	
	Non-operated	Splenectomized
Number of patients	55	45
Platelets Count (x10^3^/mm^3^)	128.4 ± 12.0	254.1 ± 10***
PT (Sec)	19.3 ± 0.63	13.8 ± 0.63***
TT (Sec)	13.7 ± 0.21	13.9 ± 0.26
PTT (Sec)	37.9 ± 1.47	26.9 ± 0.16***
Fibrinogen (g/L)	2.62 ± 0.10	2.82 ± 1.2.1
Factor II (%)	66.5 ± 2.3	71.6 ± 1.6
Factor VII (%)	49.9 ± 2.5	65.0 ± 2.4***
Factor VIII (%)	90.5 ± 4.1	106 ± 6.3 *
Factor IX (%)	59.9± 2.4	82.7 ± 4.3***
Factor X (%)	63.7 ± 3.6	77.7 ± 3.7**
Protein C (%)	65.6 ± 2.8	77.6 ± 4.1**
Antithrombin IIa (%)	92.7 ± 3.5	96.2 ± 3.6

Values are expressed as mean ± standard error (SE) and compared by unpaired Student’s t- test

*P<0.05

**P<0.01

***P<0.0001.

**Fig 1:**
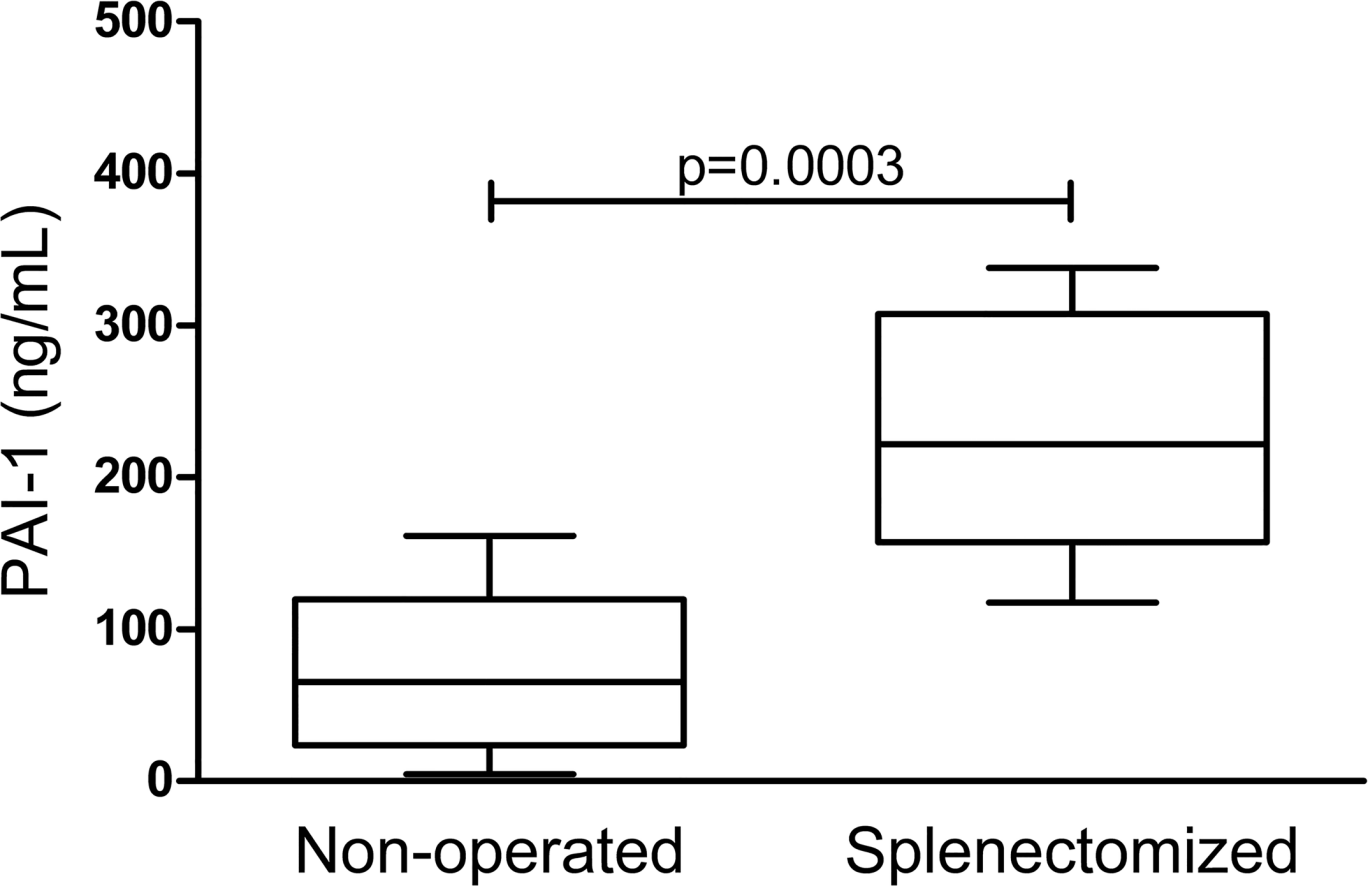
Plasma levels of PAI-1 in patients with hepatosplenic schistosomiasis who have undergone surgical splenectomy, or are non-operated. The box shows the 25^th^ to 75^th^ percentile of the PAI-1 distribution, while the horizontal bar inside the box shows the median values (65.2 vs 221.5 ng/mL). The upper and lower bars indicate the maximum and minimum values, respectively. Mann Whitney test was used to analyze the difference between the two groups.

The median plasma level of PAI-1 in splenectomized patients was 3-fold greater (221.5 vs. 65.2 ng/mL; P = 0.0003) than in the non-operated patients (Fig 1).

## Discussion

Portal hypertension associated with *S*. *mansoni* infection has a major impact on morbidity and mortality, due to the possibility of bleeding from esophageal or gastric varices [22]. Splenectomy is an established therapeutic procedure to treat and help prevent new episodes of gastrointestinal bleeding; the marked reduction of pre-sinusoidal portal hypertension improves hemodynamic abnormalities [12]. In this study we have measured markers of liver function and blood coagulation parameters in HS schistosomiasis patients, comparing those who have undergone splenectomy with a non-operated group.

Recently, we reported that in HS schistosomiasis the common indicators of liver injury ALT, AST, ALP and bilirubin are twice the levels of those in healthy individuals, while γGT is five-fold higher [15]. Mechanisms proposed to explain elevated ALP and γGT in HS patients, include the compression of small intrahepatic bile ducts by schistosomal granulomas [23], though this mechanism was not confirmed by Amaral et al. [24] who found no changes in intra or extra-hepatic biliary tracts by ultrasound examination. Our previous study also demonstrated that levels of yGT increase with progression of PPF, suggesting that this enzyme is a useful marker for stratifying the different patterns of PPF [15]. The severity of PPF reflects host immunogenic response and degree of infection, which amplify splenic volume and consequently increase hyperflux in the spleno-portal region. These factors in combination raise portal hypertension [11–13].

In the present study, although both groups of HS schistosomiasis patients had similar age, gender and pattern of PPF, we still found a significant reduction in the portal vein diameter of the splenectomized patients, reflecting decreased portal hypertension following surgery. There were also significant differences in some liver function tests between our two patient groups; serum levels of bilirubin and ALP were decreased in splenectomized patients, whereas γGT levels were similar. Previous studies have demonstrated that the greater the portal blood flow the higher the levels of ALP and γGT, findings linked to possible anatomical changes in the biliary tree caused by fibrosis in the portal region [25]. Additionally, serum ALP is higher in schistosomiasis patients with portal hypertension than those without, although no differences are noted in γGT levels [26]. Hence, we conclude that our finding of high serum ALP is related to portal hypertension insofar as splenectomy reduces the portal flow, which in turn decreases ALP levels. In contrast, the elevation of γGT in schistosomiasis is probably associated not only with portal hypertension but also with advanced PPF.

The second indicator of improved liver function in our splenectomized patients was their lower serum levels of bilirubin (total, direct and indirect) compared to non-operated HS schistosomiasis patients. The low indirect bilirubin fraction in splenectomized patients could result from reversal of the accelerated hemolysis that occurs in the splenic parenchyma due to hypersplenism. Furthermore, the reduction in direct bilirubin could reflect a better synthetic capacity of hepatocytes following the decrease of portal pressure in our splenectomized patients. Indeed, Toledo et al. [26] also observed lower total and direct serum bilirubin in schistosomiasis patients without portal hypertension compared to those with.

Serum albumin levels in our two patient groups were within the normal range, and no increased tendency was evident in splenectomized patients. As the diseased liver in schistosomiasis largely preserves hepatocyte architecture and synthetic capacity, it is perhaps not surprising that albumin levels were unchanged. However, another liver-secreted protein, lecithin-cholesterol acyltransferase (LCAT) [27], is considered a more sensitive serum test of hepatocyte synthetic capacity [28,29] and in a previous study we showed that splenectomy significantly improved low plasma LCAT activity by around 50%, compared to non-operated HS schistosomiasis patients [30]. This finding supported an earlier proposal that LCAT assay might be a useful test in schistosomiasis mansoni for assessing disease severity [31].

Although hepatocyte synthetic capacity is largely conserved in schistosomiasis, some patients in an advanced stage of the disease have hemostatic abnormalities and altered mechanisms of fibrinolysis [14–16]. In the present study, we observed prolongation of the PT and PTT in the non-operated patients with HS schistosomiasis. However, the splenectomized group had no increases in PT and PTT, suggesting that when prolongation occurs it reflects not only hepatocyte synthetic capacity, but also the degree of portal hypertension. Our earlier study found that the changes in PT, PTT and TT in HS schistosomiasis were more pronounced with disease progression [15]. Here, we also noted slightly higher fibrinogen levels in the splenectomized patients compared to the non-operated group, although this difference was not significant. The prolongation of PT, PTT and TT as well as hypofibrinogenemia is a well-established finding in patients with schistosomiasis [16].

Reduced levels of factors VII, IX, X, and protein C have also been reported in human schistosomiasis [16]. Indeed, we found lower levels of factors VII, VIII, IX, X, protein C in the non-operated patients with HS schistosomiasis compared with the splenectomized group (Table 3). It is assumed that the reduction of vitamin K in HS schistosomiasis patients is caused by impaired hepatic synthesis or increased consumption of these coagulation factors, [15,16]. Our present findings suggest that the lower portal pressure after splenectomy will improve hepatic synthetic capacity and reduce consumption of the factors.

An additional finding, potentially significant, from our study relates to low levels of PAI-1 in HS schistosomiasis, highlighted by El-Bassiouni et al. [18] and ourselves [15]. There is evidence that PAI-1 is synthesized by endothelial cells [32], as well as hepatocytes [33–34]. Thus, the higher level of serum PAI-1 in our splenectomized patients suggests that the reduction of portal pressure may not only improve hepatic synthetic function, but also the generation of PAI-1 by endothelial cells. Therefore, this possibility and other functional properties of endothelial cells merit further investigation.

One limitation of our study is that it was conducted at a single hospital, the Hospital das Clínicas, UFPE. This is the reference hospital for schistosomiasis in Pernambuco State and receives the most severe cases of schistosomiasis, usually patients with a history of one or more episodes of gastrointestinal bleeding and hence a high proportion with abnormal liver function tests. Furthermore, we had no information on liver function and blood coagulation parameters in HS schistosomiasis patients before they had undergone splenectomy, due to the wide time frame around 11.5 years post-splenectomy; hence, they were compared with a non-operated group of patients with HS schistosomiasis. Therefore, the findings from the present study may not extrapolate to all patients from endemic areas who present with the HS form of schistosomiasis mansoni.

In summary, we conclude that the abnormal changes observed in liver function tests and components of hemostasis in non-operated HS schistosomiasis are less severe, or not present, in splenectomized patients. This implies that portal hypertension is an important factor in the pathogenesis of the liver fibrosis and hemostatic dysfunction observed in human HS schistosomiasis mansoni. Moreover, a key point arising from this study is that splenectomy ameliorates liver function tests in patients with the most severe form of schistosomiasis; this may eventually reduce clinical symptoms and perhaps prolong life, a significant possibility as, in general, cure of HS schistosomiasis mansoni is not possible.
